# Year-Round Bat Activity and Species Richness Near Temporary Ponds in the Mediterranean Region

**DOI:** 10.3390/life13071495

**Published:** 2023-07-01

**Authors:** Ioanna Salvarina, Panagiotis Georgiakakis, Artemis Kafkaletou Diez, Triantafyllia-Maria Perivolioti, Ioanna Vassilaki, Matina Kalcounis-Rueppell

**Affiliations:** 1 Laboratory of Ichthyology, School of Biology, Aristotle University of Thessaloniki, University Campus, P.O. Box 134, 54124 Thessaloniki, Greece; triaperi@bio.auth.gr (T.-M.P.); jeannettevas@windowslive.com (I.V.); 2 Natural History Museum of Crete, University of Crete, 71305 Irakleion, Greece; pangeos@nhmc.uoc.gr; 3 Independent Researcher, 73134 Chania, Greece; artemisdiez@hotmail.com; 4 Department of Biological Sciences, University of Alberta, Edmonton, AB T6G 1Z2, Canada; kalcounis.rueppell@ualberta.ca

**Keywords:** Chiroptera, ephemeral ponds, Greece, hydroperiod, acoustic monitoring, habitat type 3170

## Abstract

Mediterranean temporary ponds are recognized as conservation priority habitats that face anthropogenic threats and are important habitats for a number of aquatic and terrestrial animals and plants. Bats are a diverse group of animals that use ponds for drinking and feeding on emerging aquatic insects and terrestrial insects in the riparian zone. We investigated the importance of temporary ponds for bats in Greece by acoustically sampling bat community structures and activity at temporary ponds throughout the year. We sampled monthly, from 3 to 13 months in 2019–2020, at sites at the pond edge and approximately 150–300 m away from the edge, at four temporary ponds in northern and southern Greece. Our results confirm the importance of temporary ponds for bats as activity was recorded year-round and was high in all but the winter months. In general, the distance to the edge of the pond and the presence of water in the pond explained bat activity together with air temperature. Importantly, whether dry or not, all ponds supported bat activity, independent of their particular characteristics. This study highlights the urgent need for the conservation of temporary ponds, especially in areas with limited water availability.

## 1. Introduction

Freshwater bodies are necessary for bats (Chiroptera) for both drinking and feeding, in particular for species that feed on emerging aquatic insects or fish (reviewed by [1,2]. Water is especially important for lactating bats as more drinking passes were recorded by lactating females than by non-reproductive adult females tracked in Colorado, USA [3]. Distance from water explains a large part of the variation in the use of space by bat species [4]. Water quality and quantity are threatened by anthropogenic impacts in many parts of the world, including the Mediterranean [5]. Bats face anthropogenic threats, such as habitat loss or modification, poisoning from pesticides, roost loss or disturbance and, in some parts of the world, persecution [6]. Riparian and aquatic habitat loss and/or pollution also constitute threats for bats [1]. Climate change might also result in changes in bats’ distribution and, in extreme scenarios, in losses of bat diversity [7]. The variables that are more important for the distribution of Mediterranean bat species are average temperature, relative humidity and monthly precipitation [7]. A drought event in Portugal, for example, influenced the reproduction of the free-tailed bat *Tadarida teniotis* [8]. Additionally, water body significance was shown by a strong positive effect on bat activity, especially during the lactation and post-lactation periods [9]. Wetlands are affected by temperature increases that can change their spatial distribution or water availability to the point of drying [10]. Major and minor water level decreases have occurred in lakes in the Mediterranean and other semi-arid areas due to climate change and water diversion [11].

Mediterranean temporary ponds are defined as very shallow ponds (a few centimeters deep), isolated from permanent water bodies, which undergo a periodic cycle of flooding and drought, and have a characteristic flora and fauna adapted to this alternation. They are a priority habitat type (3170 in Annex I of the Habitats Directive 92/43/EU) mainly distributed in southern European Union (EU) countries, especially in dry and sub-arid areas. However, small standing-water ecosystems, including temporary ponds, are still underrepresented in the EU Water Framework Directive and further implementation in the directive would assist their conservation [12].

Temporary ponds have been shown to be important for bats in Mediterranean countries (Israel [13]; Portugal [14]). Temporary ponds in Greece face pressures related to agriculture, urbanization and tourism, such as hydrologic disturbance and water pollution and need immediate actions for the reestablishment of their hydroperiod and water quality; e.g., [15,16,17]. Although bat communities in Greece have been studied close to lakes, e.g., [18,19,20,21], information on how bats use and depend on temporary ponds is lacking. As an example, the NATURA technical report that is focused on the management of Mediterranean temporary ponds [22] for animal species, such as birds, does not consider bats at all.

Greece has among the highest bat diversity in Europe, with 36 bat species [23] of a total of 45 in Europe. In addition to water being critical for bats, bats are recognized as bioindicators [24] and particular bat species have been suggested as ecological indicators of water quality [25,26]. We investigated the importance of temporary ponds for bats in Greece by acoustically sampling bat community structures and activity at temporary ponds throughout the year. We were interested in describing bat activity and species richness at temporary ponds throughout the year at times when the ponds were both wet and dry. We predicted that bat activity would be higher at the edge of the pond, especially when there was water present in the pond, compared to sites far from the edge of the pond. We also assessed the effect of surrounding land cover on bat activity at temporary ponds.

## 2. Methods

### 2.1. Study Sites

**Figure 1:**
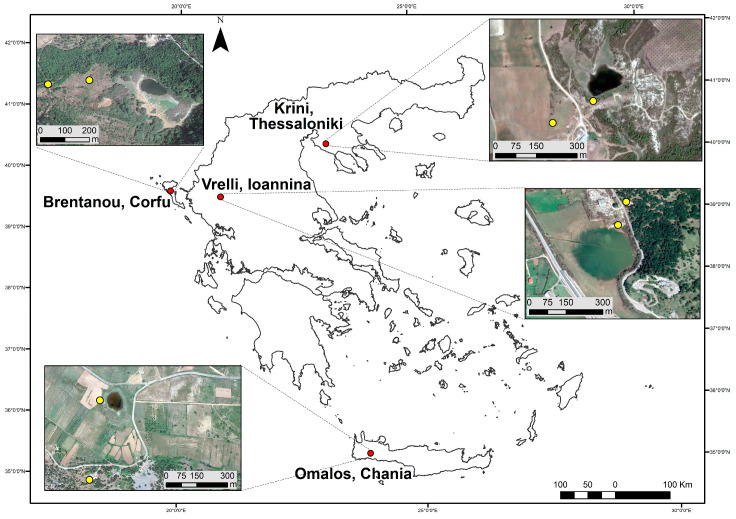
Map of Greece with the location of four temporary ponds shown (red dots). Inset: aerial photos at each pond with the two recording sites shown (yellow dots; one near the water edge when wet and one far from the edge ca. 300 m from the edge of the pond when wet).

We sampled monthly at sites at the edge of the pond and approximately 150–300 m away, at four temporary ponds in northern and southern Greece. We acoustically monitored bat activity at the temporary ponds of Brentanou (Corfu, Ionian Islands), Omalos (Chania, Crete), Vrelli (Ioannina, Epirus) and Krini (Thessaloniki, Central Macedonia) (Figure 1). We only selected freshwater temporary ponds and excluded saline or semi-saline ponds. Although saline and semi-saline ponds are abundant in Greece, bats do not use these for drinking, and thus, the results would not be directly comparable with the freshwater ponds.

Brentanou pond (also known as Bertzanos; henceforth Brentanou) is on Corfu island at an elevation of 45 m and has temporary water coverage [27]. Brentanou usually covers a surface area of 6.160 ha and is a Tp Ramsar Type [Permanent freshwater marshes/pools; ponds (below 8 ha) defined as marshes and swamps on inorganic soils; with emergent vegetation, water-logged for at least most of the growing season]. Brentanou is nationally protected (Presidential Decree, Government Gazette 229/AAΠ/2012).

Krini pond (also known as Krini Inland Marsh or Monopigado pond; henceforth Krini), at an elevation of 243 m, is a Ts Ramsar type and covers a 0.440 ha surface area [28]. It usually has water at the end of winter and is dry in summer. Our study year was exceptional as this pond was dry until the end of April due to low rainfall in the winter from 2019 to 2020.

Omalos pond (henceforth Omalos) is located in Omalos plateau at 1060 m above sea level, within the boundaries of the Natura 2000 site ‘Lefka Ori kai paraktia zoni’ (GR4340008) and is nationally protected (Presidential Decree, Government Gazette 229/AAΠ/2012). Omalos is a Ramsar type Ts (seasonal/intermittent freshwater marshes/pools on inorganic soils; includes sloughs, potholes, seasonally flooded meadows, sedge marshes). Omalos, when inundated, covers a surface of 0.790 ha [29]. Omalos usually has water or ice in the winter months and can completely dry before the first rain in autumn, after which it fills again. There is a known cave used as a roost site located about 5 km from Omalos with the occasional presence of 11 species, but mostly in small numbers (<5 ind) [20]. The highest numbers noted were several individuals of *Myotis capaccinii* in August 2008 and about 40 individuals of *Miniopterus schreibersii* in October 2006 [20].

Vrelli pond, (henceforth Vrelli) near the city of Ioannina, at an elevation of 485 m, has a surface of 5.830 ha (the largest water-covered surface, as measured during our study). It is usually full in autumn and dry in spring. Additional information for Krini and Vrelli ponds is limited.

Each pond differed in terms of the timing during which it was dry versus when it was wet. Vrelli was dry from the end of spring until the end of autumn. Krini was dry from the beginning of summer until the beginning of the next spring. Omalos was only dry in October and November, inundated in December, and then froze in January and February. Brentanou had water throughout the recordings.

### 2.2. Bat Acoustic Monitoring

Bat activity was assessed with acoustic monitoring during 3 (or occasionally 2 or 4) consecutive nights per month at each pond. At each pond, we recorded at two sites; one site was “close” to the edge of the pond and the other was “far”, at roughly 150–300 m distance from the edge. We recorded over 12 consecutive months at Vrelli (March 2019–February 2020), 13 consecutive months at Krini (June 2019–June 2020) and 11 consecutive months at Omalos (March 2019–April 2020). We were interested in assessing whether there is also any winter activity. Although bats mostly hibernate in winter in Greece and they are more active from March to November, low bat activity has been recorded also during winter (own unpublished data). During our study, there was snow in the area of the Omalos pond and it was frozen during January and February 2020. We did not have access to Omalos during this time so it was not possible to set up our equipment during these months. Due to practical difficulties, recordings were only conducted for 3 months (March, April and the beginning of June 2019) at Brentanou. The “close” site was 1–2 m from the water (from the edge when the pond was full) and the “far” site was approximately 150 m away at Vrelli and Krini, and approximately 300 m away from water at Brentanou and Omalos. The difference in the distance between the sites far from the water was related to site access and security for the equipment.

**Figure 2:**
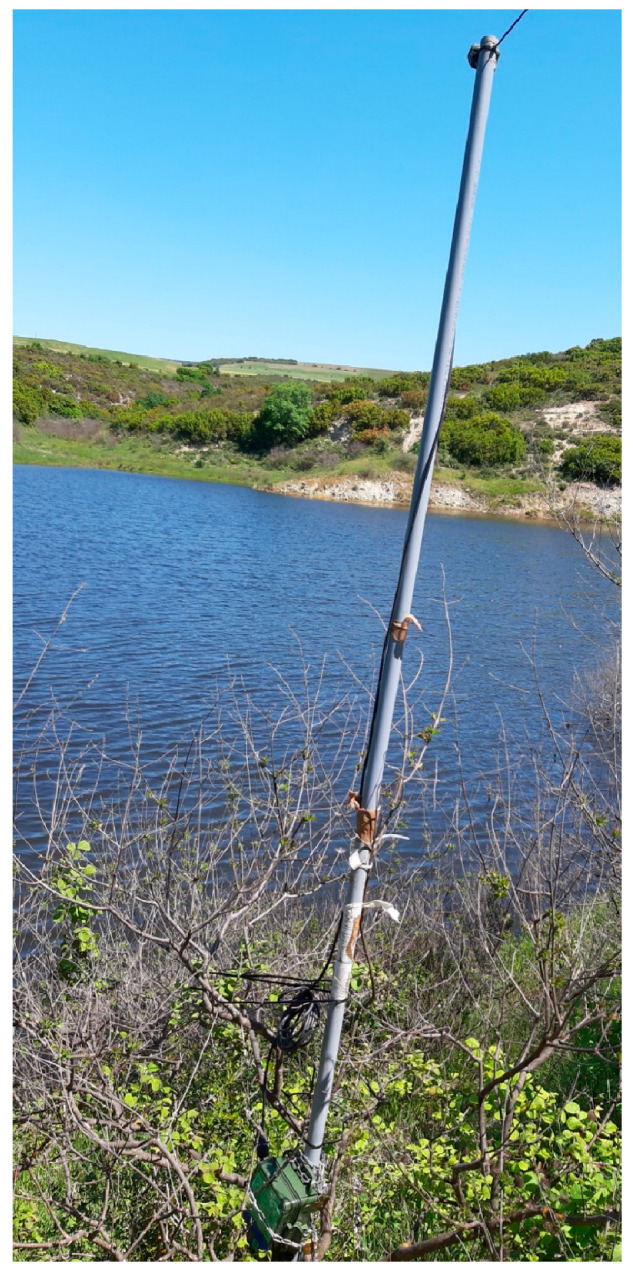
An example of how the SM4BAT recorder and the microphone were placed at the site Krini, close to the water. The recording unit is green and at the bottom of the pole and the microphone is wired to the top of the pole. The microphone is facing the water.

Acoustic sampling started 30 min prior to sunset and continued through the night until sunrise using SM4BAT-FS (Wildlife Acoustics, Maynard, MA, USA) bat recorders with the following settings: gain: 12 dB, 16 k high filter: on, sample rate: 256 kHz, min duration: 3 ms, max duration: none, min trig freq: 16 kHz, trigger level: 12 dB, trigger window: 3 s, max length: 00 m:15 s. We used SMM-U2 microphones (Wildlife Acoustics) connected to recorders attached to the top of 3 m poles (Figure 2) at least 1 m away from vegetation and buildings. The microphone was always facing toward the sky and the back of the microphone was opposite the lake to optimize its cardioid sensitivity pattern. Bats usually stop hunting when it rains [30,31] and therefore we avoided, as much as possible, recording when there was rain or/and strong wind.

### 2.3. Acoustic Analysis

Recorded sound files were organized into recording sessions (one session for three consecutive nights at each site, each month), were searched for bat echolocation and social calls with the software bcAdmin (Versions 3.6.24 and 4) and bat echolocation calls were automatically identified with the batIdent (Version 1.5) trained for European bat species (both software from EcoObs GmhH, Nuremberg, Germany). BatIdent identifies each sequence on a species or group level with a probability of correctness. Since automated species identification carries a risk of misidentification [32,33], a number of recordings and their identifications were manually confirmed using bcAnalyse 3 Pro Standalone (EcoObs GmhH, Nuremberg, Germany) by three of the authors (IS, PG, AKD). Manual identification was based on the Greek Bat Call Library, developed by Papadatou [19,34], Georgiakakis [21,35] and Kafkaletou-Diez [36] and maintained in the Natural History Museum of Crete, University of Crete, Greece. Additionally, published articles on the bat calls of adjacent regions [37,38] were used in cases where identification was difficult. All files that were identified as empty by BatIdent were also manually checked for quiet bat calls or calls obscured by ambient noise and were identified where possible. All sequences automatically identified as *Plecotus* spp. or *B. barbastellus* were also manually confirmed, since they were often misidentified.

For each recording session, a list of the recorded species was made. Although the calls of *Myotis* species can be often attributed to species groups, the correctness of identification is often compromised by a variety of factors (e.g., call amplitude; [34,37]). Therefore, calls were grouped as “*Myotis* spp.” as in [39] and [40]. Similarly, calls of *Plecotus* species were grouped as “*Plecotus* spp.”.

In mainland Greece, *Pipistrellus nathusii* and *P. kuhlii* were grouped as “Pipistrelloids middle frequency”, unless identification could be aided by the presence of social calls [41,42,43]. On Crete, *P. nathusii* is extremely rare and has not been reported for the broader area surrounding Omalos [20], therefore, it was not considered in this study. *Hypsugo savii* often has calls with lower frequencies, but in many cases, it overlaps with *P. nathusii* and *P. kuhlii* [37] and cannot be discriminated when social calls are absent [44]. In these cases, when identification to species level was not possible, calls with an end frequency of 34–37 kHz were classified as “Pipistrelloids low frequency”. For simplicity and since these species often overlap, *Hypsugo savii*, *P. nathusii* and *P. kuhlii* were grouped together for analyses.

*Pipistrellus pipistrellus* is one of the most common bat species in Greece [18], but on Crete, it is replaced by *P. hanaki*, with echolocation calls of the two species being almost identical [20]. Discrimination of these two pipistrelles from *Miniopterus schreibersii* was not possible when the end frequency exceeded 49 kHz and social calls were not available. Similarly, discrimination between *M. schreibersii* and *Pipistrellus pygmaeus* was not possible when the end frequency ranged between 56 and 59 kHz and social calls were absent [21,34,35,45]. Echolocation calls with end frequencies in these areas of overlap were classified as “Pipistrelloid high frequency” if no social calls were available.

High-frequency Rhinolophids (*R. hipposideros*, *R. mehelyi*, *R. euryale*) live in sympatry in mainland Greece and it is not possible to identify their calls due to high overlap in call characteristics [36]. In these cases, the calls were classified as “*Rhinolophus* high frequency”.

Calls with an end frequency between 23 and 30 kHz were classified as Nyctaloid, since discrimination between *Eptesicus serotinus* and *Nyctalus leisleri* and *N. noctula* is difficult [37], especially in Greece [34,36]. When the end frequency ranged between 19 and 23 kHz, calls were identified as *N. noctula*. *Vespertilio murinus* is quite rare on mainland Greece, but the recording of its echolocation calls cannot be ruled out. It is possibly absent from Crete and Corfu. All calls with an end frequency between 10 and 19 kHz were identified as *T. teniotis* [34,36]. In very few cases, our recorded call quality was low and, in those cases, calls were classified as “*Chiroptera* sp”.

Since the parameters of bat calls for several species overlap, and in order to avoid false identification, we grouped species identifications into the following groups for our analyses:

- *Myotis* spp.
- *Plecotus* spp.
- *Barbastella barbastellus*
- Pipistrelloid middle and low frequency = *P. nathusii*, *P. kuhlii*, *H. savii*
- Pipistrelloid high frequency = *P. pipistrellus*, *P. pygmaeus*, *P. hanaki*, *M. schreibersii*
- *P. hanaki*
- *Rhinolophus ferrumequinum*
- other *Rhinolophus* spp. (one or more of the species: *R. blasii*, *R. hipposideros*, *R. mehelyi*, *R. euryale*)
- Nyctaloid (*Nyctalus* spp., *Eptesicus* spp., *Vespertilio murinus*, *Tadarida teniotis*)
- *Tadarida teniotis*
- *Chiroptera* sp.

Bat activity was reported as the number of sequences per hour of recording (sequences/hour) across a recording session. A sequence is equivalent to a bat pass and is defined as ≥2 bat calls characteristic of bat echolocation. The mean value of sequences/hour was calculated across the three nights.

### 2.4. Meteorological Data

Mean temperature, wind speed (km/h) and precipitation (mm) were estimated for each recording session (across the three consecutive nights) for each pond. Although we tried to avoid rain and strong wind, we could not completely exclude it for all three consecutive nights of the recordings. Meteorological data were taken from the site of the National Observatory of Athens (IERSD/NOA) (https://www.meteo.gr/ last access date 1 September 2020). For each pond, meteorological data were acquired from the closest meteorological NOA station. For Vrelli, Brentanou, Krini and Omalos, data were acquired from the stations of Ioannina, Kerkyra, Polygyros and Samaria-Xyloskalo, respectively.

### 2.5. Landscape

Spatial analyses were performed using the Geographic Information System (ArcGIS 10.3; ESRI) with Corine Land Cover (2012) across the study sites. The percentage of first-level land cover for landscape elements was calculated using a buffer zone of 500 m, 1 km and 5 km radius, around each site. The first buffer zone (500 m) was selected to search for small-scale impacts and was only calculated for the site close to water. The second buffer zone (1 km) was selected because this zone is meaningful for European bat activity and relevant for landscape management [46], and was separately calculated for each site (close and far). The third buffer zone (5 km) was chosen to cover the landscape relevant for bats that fly several kilometers per night [26] and was calculated for the sites close to water but was used in the analysis for both sites as the distance between the sites is very small within this buffer zone. Buffer zones were intersected with the Corine Land Cover features and the areas of the intersections were expressed as the percentage of the total buffer zone, in order to represent the total land cover percentage for each land cover type.

The land cover types were, and included level 2 according to the Corine land cover nomenclature types, as follows: artificial areas (including industrial or commercial units, public, industrial or mine dump sites, construction sites, discontinuous urban areas, port areas, sport and leisure areas, road and associated land), agricultural areas (non-irrigated arable land, olive groves, land principally occupied by agriculture, with significant areas of natural vegetation, complex cultivation patterns, vineyards), forest areas (sclerophyllous vegetation, coniferous forest, natural grasslands, mixed forest) and water bodies (sea and ocean).

### 2.6. Data and Statistical Analysis

Nightly and monthly activity (sequences/hour) was plotted for each pond combining both close and far sites. The relationship between bat activity and both the meteorological parameters and type of landscape cover was investigated using linear regression (Pearson’s correlation). General linear models (GLMs) were constructed for each pond to investigate how the season (defined as winter: December–February, spring: March–April, summer: June–August, autumn: September–November), the distance from water (close and far from pond edge) and the presence of water at the pond (yes or no) explained variance in total bat activity. Models were chosen based on the Akaike information criterion (AIC). Bat activity was compared between the close and far sites in each season at each pond using a Mann-Whitney test. The same models and tests were repeated for species richness. All analyses and plots were performed in R version 3.0.0 (R Core Team 2013) [47], in RStudio version 1.1.38 (RStudio Team 2016) [48] and additionally using the packages ggplot2 (Wickham 2016) [49] and gridExtra (Auguie 2012) [50].

## 3. Results

In total, we had seventy-eight recording sessions across eight sites (four ponds) that corresponded to two hundred and fifty-two full nights of bat recording (mostly three nights per session, but occasionally two or four; Table 1). Overall, 78,261 sequences were recorded, belonging to eleven species, three genera and one species group from four families (Vespertilionidae, Rhinolophidae, Molossidae, Miniopteridae; Table 2). Actual species richness was likely higher because *Myotis*, *Plecotus*, *Rhinolophus* and Nyctaloids were possibly present with more than one species (Table 2). Species composition was similar at Vrelli, Krini and Brentanou, whereas it differed at Omalos, especially with respect to the presence of *Pipistrellus* species (Table 2). *Barbastella barbastellus* was only found at Vrelli (Table 2).

The group with the highest contribution to activity (37.5% of total activity) was Pipistrelloids with low frequency (*Pipistrellus kuhlii/nathussi* and *H. savii*). Considerable activity was also attributed to *T. teniotis* (27.2% of total activity), which had the highest contribution to total activity at Omalos (up to 94.6% in October, at the close site), followed by *P. hanaki* (up to 63.9% in September, at the far site).

The highest bat activity was recorded close to the water at Omalos in June 2019 (568.100 sequences/hour), while the lowest was close to zero (0.067 sequences/hour) and was from the far site at Krini in January 2020. Very low bat activity was recorded at mean temperatures as low as 2 °C (0.147 sequences/hour, Vrelli, January 2020).

Total bat activity and species richness moderately increased with temperature across all ponds (Pearson’s test S = 34,457.560, *p* < 0.001, rho = 0.564 and S= 37,605.570, *p* < 0.001, rho = 0.525, respectively). Bat activity increased with temperature at each pond (Krini: S = 820.040, *p* < 0.001, rho = 0.720; Vrelli: S = 563.090, *p* < 0.001, rho = 0.755; Omalos: S = 559.230, *p* < 0.001, rho = 0.684) except at Brentanou (S = 22.450, *p* = 0.485, rho = 0.359), where only a few sequences were recorded. At each pond, species richness increased with temperature (*p* > 0.001). Across all ponds, bat activity was not related to wind speed (*p* > 0.480). Species richness was correlated, negatively, with wind speed only at Vrelli (S = 3360.700, *p* = 0.023, rho = −0.461). Across all ponds, bat activity decreased with rain (S = 102510.500, *p* =0.008, rho= −0.296) and the same was true at Vrelli (S = 3857.400, *p* < 0.001, rho = −0.677) and Omalos (S = 2658.600, *p* = 0.018, rho = −0.501). Species richness decreased with rain across all ponds (S = 100,568.900, *p* = 0.016, rho = −0.272), at Vrelli (S = 3484.200, *p* = 0.010, rho = −0.515) and at Omalos (S = 2566.500, *p* = 0.036, rho = −0.449).

At Krini, the season (*p* = 0.006 for summer and *p* = 0.038 for autumn) and water presence (*p* = 0.008) best explained variation in bat activity, whereas temperature (*p* < 0.001), rain (*p* = 0.008), water presence (yes or no) (*p* = 0.009) and proximity to water (*p* = 0.014) best explained the variation in species richness. At Omalos and Vrelli, temperature best explained variation in bat activity (*p* < 0.001). Proximity to water, additionally, explained bat activity at Omalos (*p* = 0.034). Species richness was explained by temperature and season (summer, *p* = 0.030) at Omalos, and by season (winter, *p* = 0.001) at Vrelli. At Brentanou, temperature (*p* = 0.009) explained species richness, whereas no variables explained bat activity (Supplementary Material).

When all ponds were included in the same model, the model that best explained activity included the pond, season, temperature and proximity to water (close vs. far). In this model, pond (Omalos *p* = 0.001) explained most of the variation followed by distance from water (*p* = 0.015) and mean temperature (*p* = 0.024) (Supplementary Material). The model that best explained species richness included the pond, season, temperature, proximity to water (close vs. far) and water presence, with variability being explained by the pond (Omalos, *p* < 0.001, Vrelli, *p* = 0.005), temperature (*p* < 0.001) and water presence (yes or no) (*p* = 0.039) (Supplementary Material).

**Figure 3:**
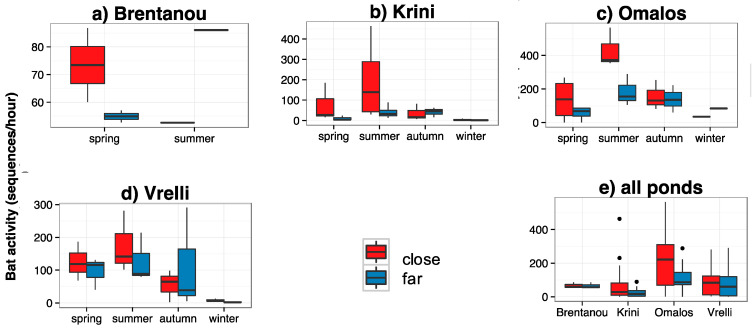
Total bat activity (sequences/hour) at the sites close and far from water, (**a**–**d**): per temporary pond and per season and (**e**): per pond, for all seasons together. The recordings were conducted monthly, during three consecutive, full nights monthly over three months (March, April and beginning of June 2019) at Brentanou, over twelve consecutive months (March 2019–February 2020) at Vrelli, thirteen consecutive months (June 2019–June 2020) at Krini and eleven consecutive months (March 2019–April 2020) at Omalos. Close: at the water’s edge, far:150–300 m from the shore.

**Figure 4:**
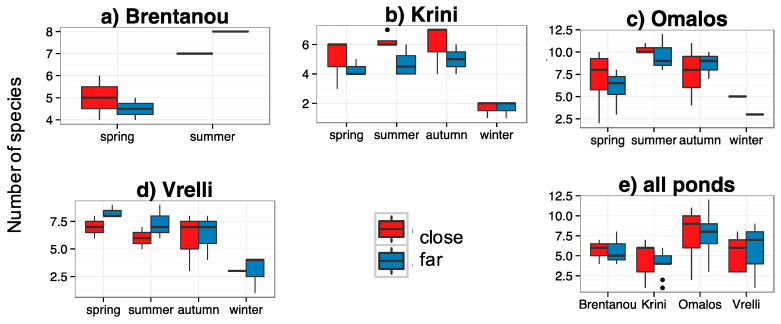
Total number of species at each site (close vs. far from water), (**a**–**d**): per temporary pond and per season and (**e**): per pond, all seasons together. Note: only confidently identified species were calculated, the actual number of the species is expected to be higher. Recordings during three consecutive, full nights, monthly over three months (March, April and the beginning of June 2019) at Brentanou, over twelve months (March 2019–February 2020) at Vrelli, thirteen months (June 2019–June 2020) at Krini and eleven months (March 2019–April 2020) at Omalos. Close: at the water’s edge, far: 150–300 m from the shore.

Bat activity was often higher at sites close to water across seasons and ponds (e.g., at Brentanou in spring, at Krini and Omalos in summer; Figure 3), but without being statistically different (in all pairs *p* > 0.200). Species richness was in some cases higher close to water (e.g., in summer and autumn at Krini), but not statistically different (in all pairs *p* > 0.060) (Figure 4).

**Figure 5:**
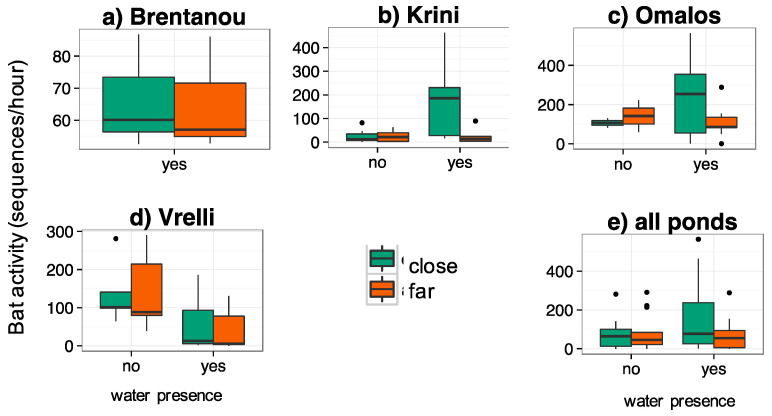
Total bat activity (sequences/hour) at each site (close vs. far from the water), per pond and for all ponds together, for the period when there was water presence or not. Recordings during three consecutive, full nights, monthly over twelve months (March 2019–February 2020) at Vrelli, thirteen months (June 2019–June 2020) at Krini and eleven months (March 2019–April 2020) at Omalos. Close: at the water’s edge, far: 150–300 m from the shore.

Comparing the sites close and far from the water line, separately at each pond for the period when they had water versus the period when they were dry, there were no statistical differences found either for bat activity (Figure 5) or for species richness. However, there was a trend for higher activity close to the water when the pond had water at Omalos and Krini (Figure 4); the same was true for species richness (not shown here). Bat activity was recorded throughout the night at all ponds regardless if they were dry or had water.

Total bat activity was positively related to artificial cover in the 1 km buffer (S = 48330, *p* < 0.001, rho = 0.389) and 500 m buffer (S = 6691.1, *p* = 0.045, rho = 0.323) and to forest cover in the 5 km buffer (S = 49,954.100, *p* < 0.001, rho = 0.368), and negatively related to agricultural cover in the 5 km zone (S = 108,203.900, *p* < 0.001, rho = −0.368). Within species or groups, there were some additional relationships. For instance, in the 5 km buffer, *T. teniotis* activity decreased with artificial (S = 105,768.500, *p* = 0.005, rho = −0.338) and agricultural cover (S = 128,139.700, *p* < 0.001, rho = −0.620) but increased with forest cover (S = 30,018.300, *p* < 0.001, rho = 0.620), while the opposite was true for the 1 km and 500 m buffer zones (Supplementary Material, Tables S2–S4).

## 4. Discussion

Our work is the first to systematically examine bat responses to changes in water availability at temporary ponds in Greece and allows us to support the suggestion of bat species to serve as bioindicators for this fragile, yet important, system. We presented the monthly activity of bats at four temporary ponds in Greece and documented year-round activity, showing that although bats respond to the presence of water at some ponds, activity levels and the number of species are high even when the ponds are dry. Ponds supported high levels of bat diversity with eleven species and four species groups recorded.

Together with temperature, distance from water and presence of water were important in explaining bat activity and species richness. At Omalos, we found support for higher levels of activity close to the edge of the pond when compared to those far from the edge. At Krini, we found higher levels of activity in both spring and autumn when there was water present. At Krini, we additionally found higher species richness associated with both nearness to the pond edge and the presence of water. We did not find support for nearness to the pond edge or availability of water at Vrelli, likely due to high variability in the data collected. Brentanou had limited sampling and had water present across all samples. Our results at Omalos and Krini are consistent with other studies in the Mediterranean region. In Spain, higher bat activity and richness were also found close to ponds than in forest areas [51]. In Israel, bat activity and diversity were found to increase with pond size [13]. Although we did not examine the effect of pond size, higher activity was noted at Omalos, which was not the largest pond, probably due to the lack of other water sources in the region. Bats also likely visit Omalos from further away, probably from the cave located about 5 km away, which indicates its importance as a water source in an arid area especially in light of the climate crisis. Temporary ponds in another Mediterranean area (Morocco) are expected to increase their water deficit by 16–67% by 2100 under the RCP8.5 climate projection scenario [52].

The strong relationship that we found between both bat activity and bat species richness and mean temperature is not surprising. Temperature and seasonality are known to be important determinants of bat activity; e.g., [53,54]. Insect densities also likely play a role as most species include aquatic or riparian insects in their diets and especially some of the bat species that contributed the highest in total bat activity, such as the *Pipistrellus* species (*especially P. nathusii* and *P. pipistrellus*) that feed on the aquatic species of non-biting midges (Chironomidae) [23]. We did not find strong relationships between total bat activity and land cover types, suggesting that the scale at which bats are responding to land cover type, if at all, is not captured by the size of our buffer zones or the land cover type resolution. An alternative is that other factors are influencing bat activity and species richness. In our species and species group analysis, we did find some land cover relationships, but these were not consistent across buffer zones.

The group *P. kuhlii/P. nathusii/H. savii* together with *T. teniotis* were the most common species contributing to total bat activity. *Pipistrellus kuhlii* was found at all of our ponds and is one of the most abundant species in Greece [18]. An acoustic study in Patras, Greece, showed 70% of total bat activity was attributed to this species [39]. In contrast, *P. hanaki* was only found at Omalos (as it is only present in Crete), while Nyctaloid species (*Nyctalus* spp. or *Eptesicus* spp.), *Myotis* spp., *Rhinolophus ferrumequinum* and *T. teniotis* were recorded at all ponds. The high prevalence of *T. teniotis* at Omalos is not surprising as it is the most abundant species in Crete [55], known to be present at all elevations and different types of landscapes including near freshwater [21].

Interestingly, total bat activity was consistent overnight in all months at all ponds, except during the winter months at Vrelli. Our results underscore the importance of temporary ponds as feeding or/and drinking sites for bats throughout the year, independent of whether water is present at the pond. Even with limited water availability, a temporary pond is likely attractive for bats as a feeding source, as insect larvae, such as Chironomidae, can be still present during the drying phase [56]. Insect sampling at these ponds would be important in future studies. Overall, we found few differences between the ‘close’ and ‘far’ from pond sites. It is likely that our ‘far’ sites were not far enough and, on the scale of a flying bat, the sites may have been both associated with a pond. Previous data collected during September (2013) from the broad region of Thessaloniki (northern Greece) show that total bat activity is almost double at sites close to water compared to those without water [57]. In particular, on average, 41 passes (13–90 passes) were recorded during 2 km transects of active recording at sites without the presence of water, while 106 passes (82–140 passes) on average were recorded at transects near lakes or rivers [57]. This suggests that our ‘far’ sites were likely not perceived by bats as being sites without water. Future studies should consider the control sites much further from temporary pond edges, and without water.

Bats were active throughout the year. This was true for all study sites, no matter the altitude and geographical position (except at Omalos when covered with snow and recordings were not conducted). This indicates that bats in the Mediterranean region are also active in winter, presumably due to the moderate temperatures (as in [58], in the coastal plain of the southeastern USA). Winter bat activity at wetlands has also been reported by Mas et al. in Spain [59]. However, whether enough food resources are available is not known. Given the trend of increasing winter temperatures [60], a possible earlier awakening of bats that usually hibernate during the winter in temperate zones is to be expected. However, if food is not yet adequate, this might have further consequences for reproductive success and survival. For species that migrate during winter, the trend of increasing winter temperatures is also worth consideration from the perspective of cues and food availability for the timing of migration and return. We recorded bat activity at temperatures as low as 2 °C. Although few studies have monitored bats during winter, bat activity has also been recorded in winter months, at a temperature as low as −7 °C, in North Carolina, USA, a region at a similar latitude with Greece [54], and at above 4.6 °C (at sunset) in another Mediterranean region (North Portugal [61]). Winter bat activity has also been recorded with temperatures above 6 °C in areas more northern than Greece, specifically in Bavaria, Germany [62]. Overall, we advocate for more studies of the winter biology of bats in Greece because we know very little about the activity patterns, nor the hibernation and/or migration patterns of those species that do not remain active.

Our study was limited by some factors. We only included four temporary ponds, even though there are at least twenty-eight Mediterranean temporary ponds across Greece [22]. Ideally, we would have sampled across all of them or a wide range of them. We likely underestimated aspects of community structures, such as species richness, because of our acoustic sampling approach where species-level identification is difficult. Future studies should include mist-netting to confirm species richness and confirm species-level responses to water availability at temporary ponds. As far as we know, there were no large roosting sites directly adjacent to the ponds other than a cave roost ~5 km from Omalos. If bat roosts were located close to some ponds but not others, this could have led to an overestimation of bat activity. Future studies should include mist-netting to confirm species richness and species-level responses to water availability at temporary ponds. Mist-netting and radio telemetry would also reveal some important aspects of winter and summer activity, including the location of hibernation and roost sites.

## 5. General Conclusions and Future Directions

Our results confirm the significance of Mediterranean temporary ponds, habitat type 3170 (Annex I of the Habitats Directive 92/43/EU), for bats in Greece. Temporary ponds are neglected habitats [63] and are important for terrestrial taxa, such as bats. High monthly bat activity and species richness were recorded at all ponds, and activity was recorded during all seasons. A better design would have included a higher number of ponds with control sites without water. Our study had some limitations, but we confirmed high levels of activity at the studied ponds and that they are important sites for bats. Our findings demonstrate the need for consideration of temporary ponds as a high priority in conservation plans for bats, and for further study of year-round use by bats at these sites. Future management and protection of Mediterranean temporary ponds, e.g., [22], should consider use by, and access to, bats throughout the year. Specific recommendations include the consideration of ensuring that temporary ponds remain accessible to bats by keeping the ponds free of vegetation, and even the construction of artificial ponds [i.e.,53] when needed.

## Figures and Tables

**Table 1:** Sample sizes per pond: recording months for the wet and the dry periods, recording nights per season, total number of recording nights and total number of sequences.

	Pond	Brentanou	Krini	Vrelli	Omalos	Total
Total number of recording months		3	13	12	11	39
Number of recording nights	Wet period	18	44	44	54	160
	Dry period	0	48	32	12	92
	Spring	10	24	18	24	76
	Summer	8	26	18	18	70
	Autumn	0	20	20	18	58
	Winter	0	22	20	6	48
Total number of sampling nights		18	92	76	66	252
Total number of sequences		5081	13,890	26,008	33,282	78,261

**Table 2:** Species and species groups confirmed (with +) at each temporary pond. * Likely, based on echolocation characteristics.

	Vrelli	Krini	Brentanou	Omalos
*B. barbastellus*	+			
*H. savii*	+	+	+	+
*Myotis* spp.	+	+	+	+
*N. noctula*	+	+	*	
Nyctaloid 1	+	+	+	+
Nyctaloid 2				*
*M. schreibersii*	*	*	*	+
*P. hanaki*				+
*P. kuhlii*	+	+	+	+
*P. pipistrellus*	+	+	+	
*P. pygmaeus*	+	+	+	
*Plecotus* spp.		+		
*R. ferrumequinum*	+	+	+	+
*R. blasii*	+	+	+	+
*Rhinolophus* high frequency	+	+	+	+
*T. teniotis*	+	+	+	+
N of species	13	13	12	11

## Data Availability

The data presented in this study are available on request from the corresponding author.

## Supplementary Materials

The following supporting information can be downloaded at: https://www.mdpi.com/article/10.3390/life13071495/s1, Table S1: The results of the selected GLM for each pond. Table S2: The results of the Pearson test between species/group activity (sequences/recording hour) and the land cover type of the 5km buffer zone. Table S3: The results of the Pearson test between species/group activity (sequences/recording hour) and the land cover type of the 1km buffer zone. Table S4: The results of the Pearson test between species/group activity (sequences/recording hour) and the land cover type of the 500 m buffer zone. Table S5: Mean activity (sequence/hour) per species/group in each season and each pond. Mean is calculated from both sites (close and far from water).
