# Supplementary material for: Year-Round Bat Activity and Species Richness Near Temporary Ponds in the Mediterranean Region
Source: Life (Basel). 2023 Jul 1;13(7):1495. doi: 10.3390/life13071495 (PMC10381810; doi:10.3390/life13071495)
Supplement: Supplementary file 1 [file life-13-01495-s001.zip › life-2378702-supplementary.pdf]

SUPPLEMENTARY MATERIAL

Table S1. The results for the selected GLM for each pond.

Selected GLM

BRENTANOU

Total activity
Call:
glm(formula = total_activity ~ TEM_MEAN, data = Brentanou)

```
Deviance Residuals:
      1        2        3        4        5        6
 22.669  -17.977   -2.880   -7.127   15.519  -10.203

Coefficients:
            Estimate Std. Error t value Pr(>|t|)
(Intercept)  54.1746    26.1973   2.068    0.107
TEM_MEAN      0.5713     1.2282   0.465    0.666

(Dispersion parameter for gaussian family taken to be 310.2779)

    Null deviance: 1308.2  on 5  degrees of freedom
Residual deviance: 1241.1  on 4  degrees of freedom
AIC: 55.019

Number of Fisher Scoring iterations: 2
```

> **Species richness**

Call:
glm(formula = N_species ~ TEM_MEAN, data = Brentanou)

```
Deviance Residuals:
       1        2        3        4        5        6
 1.04589  -0.58634  -0.45954   0.04589   0.41366  -0.45954

Coefficients:
            Estimate Std. Error t value Pr(>|t|)
(Intercept)  0.85927    1.06034   0.810  0.46318
TEM_MEAN     0.23439    0.04971   4.715  0.00921 **
---
Signif. codes:  0 '***' 0.001 '**' 0.01 '*' 0.05 '.' 0.1 ' ' 1

(Dispersion parameter for gaussian family taken to be 0.5083144)

    Null deviance: 13.3333  on 5  degrees of freedom
Residual deviance:  2.0333  on 4  degrees of freedom
AIC: 16.535
```

Number of Fisher Scoring iterations: 2

KRINI

Total activity

```
Call:
glm(formula = total_activity ~ water + close_far + season, data = Krini)

Deviance Residuals:
    Min       1Q   Median       3Q      Max
-155.528  -34.982   -4.133   28.871  234.131

Coefficients:
             Estimate Std. Error t value Pr(>|t|)
(Intercept)    -89.69      65.84  -1.362  0.18828
wateryes       163.80      55.45   2.954  0.00784 **
close_far_far  -60.83      30.76  -1.978  0.06187 .
seasonsummer   155.51      50.61   3.073  0.00601 **
seasonautumn   158.70      71.58   2.217  0.03837 *
seasonwinter   122.87      71.58   1.717  0.10151
---
Signif. codes:  0 '***' 0.001 '**' 0.01 '*' 0.05 '.' 0.1 ' ' 1

(Dispersion parameter for gaussian family taken to be 6148.352)

    Null deviance: 250486  on 25  degrees of freedom
Residual deviance: 122967  on 20  degrees of freedom
AIC: 307.79

Number of Fisher Scoring iterations: 2
```

Species Richness

```
Call:
glm(formula = N_species ~ water + close_far + season + TEM_MEAN +
    RAIN, data = Krini)

Deviance Residuals:
    Min       1Q   Median       3Q      Max
-1.65672  -0.35692   0.08154   0.38014   1.44225

Coefficients:
             Estimate Std. Error t value Pr(>|t|)
(Intercept)   -2.0712     1.8311  -1.131  0.27285
wateryes       2.0409     0.6914   2.952  0.00853 **
close_far_far -0.8435     0.3113  -2.710  0.01435 *
seasonsummer  -1.1689     0.7861  -1.487  0.15434
seasonautumn   1.4895     0.7339   2.030  0.05745 .
```

```
seasonwinter     1.6779     1.2905   1.300  0.20993   
TEM_MEAN         0.3402     0.0876   3.884  0.00109 **
RAIN             0.6133     0.2055   2.985  0.00795 **
---
Signif. codes:  0 ‘***’ 0.001 ‘**’ 0.01 ‘*’ 0.05 ‘.’ 0.1 ‘ ’ 1

(Dispersion parameter for gaussian family taken to be 0.6299133)

    Null deviance: 88.346  on 25  degrees of freedom
Residual deviance: 11.338  on 18  degrees of freedom
AIC: 70.207

Number of Fisher Scoring iterations: 2
```

OMALOS

```
Call:
glm(formula = total_activity ~ water + close_far + TEM_MEAN, 
    data = Omalos)

Deviance Residuals: 
    Min       1Q   Median       3Q      Max  
-134.09   -52.38   -12.21    55.39   244.84  

Coefficients:
              Estimate Std. Error t value Pr(>|t|)    
(Intercept)     -44.475     73.491  -0.605  0.55262    
wateryes         51.594     54.614   0.945  0.35732    
close_far_far  -96.666     42.127  -2.295  0.03399 *  
TEM_MEAN         15.264      3.531   4.323  0.00041 ***
---
Signif. codes:  0 ‘***’ 0.001 ‘**’ 0.01 ‘*’ 0.05 ‘.’ 0.1 ‘ ’ 1

(Dispersion parameter for gaussian family taken to be 9760.564)

    Null deviance: 417411  on 21  degrees of freedom
Residual deviance: 175690  on 18  degrees of freedom
AIC: 270.11

Number of Fisher Scoring iterations: 2
```

Species richness

```
Call:
glm(formula = N_species ~ water + close_far + season + TEM_MEAN + 
    RAIN, data = Omalos)

Deviance Residuals: 
     Min        1Q    Median        3Q       Max  
```

```
-2.96764  -0.91783   0.06438   1.12915   2.17798

Coefficients:
              Estimate Std. Error t value Pr(>|t|)
(Intercept)    -3.90509    2.46660  -1.583  0.13570
wateryes       -0.48406    1.87746  -0.258  0.80029
close_far_far -0.45455    0.70595  -0.644  0.53006
seasonsummer   -9.95258    4.16332  -2.391  0.03143 *
seasonautumn   -5.21232    3.13538  -1.662  0.11865
seasonwinter   -2.26166    1.33113  -1.699  0.11141
TEM_MEAN        1.11390    0.32137   3.466  0.00378 **
RAIN            0.20440    0.05842   3.499  0.00354 **
---
Signif. codes:  0 ‘***’ 0.001 ‘**’ 0.01 ‘*’ 0.05 ‘.’ 0.1 ‘ ’ 1

(Dispersion parameter for gaussian family taken to be 2.741006)

    Null deviance: 168.773  on 21  degrees of freedom
Residual deviance:  38.374  on 14  degrees of freedom
AIC: 92.673

Number of Fisher Scoring iterations: 2
```

VRELLI

Total activity

```
Call:
glm(formula = total_activity ~ water + close_far + TEM_MEAN +
    RAIN, data = Vrelli)

Deviance Residuals:
   Min      1Q  Median      3Q     Max
-92.97  -29.33   -3.79   19.22  117.64

Coefficients:
              Estimate Std. Error t value Pr(>|t|)
(Intercept)     -81.551     60.836  -1.341 0.195890
wateryes         48.586     41.363   1.175 0.254657
close_far_far    -6.260     23.903  -0.262 0.796224
TEM_MEAN          9.999      2.523   3.964 0.000833 ***
RAIN             -5.887      4.168  -1.412 0.173975
---
Signif. codes:  0 ‘***’ 0.001 ‘**’ 0.01 ‘*’ 0.05 ‘.’ 0.1 ‘ ’ 1

(Dispersion parameter for gaussian family taken to be 3428.037)

    Null deviance: 171752  on 23  degrees of freedom
Residual deviance:  65133  on 19  degrees of freedom
AIC: 269.86
```

Number of Fisher Scoring iterations: 2

Species richness

```
Call:
glm(formula = N_species ~ water + close_far + season + TEM_MEAN +
    RAIN, data = Vrelli)

Deviance Residuals:
    Min       1Q   Median       3Q      Max
-2.1885  -0.3848   0.1151   0.4886   1.9174

Coefficients:
              Estimate Std. Error t value Pr(>|t|)
(Intercept)    -62.25510   52.08128  -1.195 0.249369
wateryes        69.32330   52.74589   1.314 0.207280
close_ far_ far   0.75000    0.40457   1.854 0.082293 .
seasonsummer    67.72365   52.81066   1.282 0.217976
seasonautumn    69.14514   53.10657   1.302 0.211347
seasonwinter    -4.02252    0.95926  -4.193 0.000688 ***
TEM_MEAN         0.03523    0.07862   0.448 0.660123
RAIN            -6.80103    4.94976  -1.374 0.188382
---
Signif. codes:  0 ‘***’ 0.001 ‘**’ 0.01 ‘*’ 0.05 ‘.’ 0.1 ‘ ’ 1

(Dispersion parameter for gaussian family taken to be 0.9820517)

    Null deviance: 116.625  on 23  degrees of freedom
Residual deviance:  15.713  on 16  degrees of freedom
AIC: 75.943
```

Number of Fisher Scoring iterations: 2

ALL PONDS

Bat activity

```
Call:
glm(formula = total_activity ~ pond + season + close_far + TEM_MEAN,
    data = bats)

Deviance Residuals:
    Min       1Q   Median       3Q      Max
-150.27   -50.20    -8.20    42.85   323.81

Coefficients:
             Estimate Std. Error t value Pr(>|t|)
(Intercept)     -67.028     66.088  -1.014  0.31402
pondKrini        33.017     42.869   0.770  0.44382
```

```
pondOmalos      156.091     45.988   3.394  0.00114 **
pondVrelli       73.927     43.551   1.697  0.09411 .
seasonsummer     15.585     44.056   0.354  0.72461
seasonautumn    -29.779     31.728  -0.939  0.35124
seasonwinter     -8.727     37.026  -0.236  0.81437
close_far_far   -49.543     19.759  -2.507  0.01452 *
TEM_MEAN          7.435      3.215   2.313  0.02373 *
---
Signif. codes:  0 ‘***’ 0.001 ‘**’ 0.01 ‘*’ 0.05 ‘.’ 0.1 ‘ ’ 1

(Dispersion parameter for gaussian family taken to be 7613.352)

    Null deviance: 992939  on 77  degrees of freedom
Residual deviance: 525321  on 69  degrees of freedom
AIC: 928.93

Number of Fisher Scoring iterations: 2
```

Species richness

```
Call:
glm(formula = N_species ~ pond + season + close_ far + TEM_MEAN +
    water, data = bats)

Deviance Residuals:
    Min       1Q   Median       3Q      Max
-4.8527  -0.7681   0.1523   0.8343   2.7348

Coefficients:
               Estimate Std. Error t value Pr(>|t|)
(Intercept)    -0.25623    1.39444  -0.184  0.85475
pondKrini       0.78411    0.78956   0.993  0.32419
pondOmalos      3.88153    0.81046   4.789 9.43e-06 ***
pondVrelli      2.24732    0.77837   2.887  0.00521 **
seasonsummer   -1.07807    0.77635  -1.389  0.16947
seasonautumn    0.09711    0.63562   0.153  0.87903
seasonwinter   -1.08275    0.69338  -1.562  0.12303
close_ far_ far -0.17882   0.34697  -0.515  0.60796
TEM_MEAN        0.25965    0.05779   4.493 2.80e-05 ***
wateryes        1.04634    0.49772   2.102  0.03924 *
---
Signif. codes:  0 ‘***’ 0.001 ‘**’ 0.01 ‘*’ 0.05 ‘.’ 0.1 ‘ ’ 1

(Dispersion parameter for gaussian family taken to be 2.347583)

    Null deviance: 513.96  on 77  degrees of freedom
Residual deviance: 159.64  on 68  degrees of freedom
AIC: 299.22
```

Number of Fisher Scoring iterations: 2

Table S2 The results of the Pearson test between species/group activity (sequences/recording hour) and the land cover type of the 5km buffer zone.

	artificial			agriculture			forest			sea		
	**S**	**p**	**rho**	**S**	**p**	**rho**	**S**	**p**	**rho**	**S**	**p**	**rho**
B. barbastella	18058.0	**0.022**	0.312	23378.1	0.433	0.109	29092	0.433	-0.109	17040	**0.009**	0.351
Myotis spp.	97487.0	**0.003**	-0.333	97774.3	**0.003**	-0.337	48525.7	**0.003**	0.337	76956	0.655	-0.052
Nyctaloid all	37689.3	**<0.001**	0.485	53102.4	**0.017**	0.274	93197.6	**0.017**	-0.274	45661	**0.001**	0.376
Pipistrelloid high frequency	85805.8	0.135	-0.173	95081	**0.009**	-0.300	51219	**0.009**	0.300	87151	0.097	-0.191
Pipistrelloid low frequency	53596.7	**0.004**	0.322	62180.2	0.060	0.214	95977.8	**0.060**	-0.214	61502.2	0.051	0.222
Pipistrelloid unidentified	26993.8	0.836	-0.029	28086.4	0.612	-0.071	24383.6	0.612	0.071	29127.4	0.427	-0.110
Plecotus spp.	24178.5	**0.001**	-0.491	24178	**0.001**	-0.491	8251.5	**0.001**	0.491	na	na	na
R. ferrumequinum	83772.4	0.211	-0.145	94437.3	**0.011**	-0.291	51862.7	**0.011**	0.291	79396.1	0.463	-0.085
Rhinolophus other than R.fer	70732.2	0.777	0.033	82592.8	0.266	-0.129	63707.3	0.266	0.129	73356.5	0.981	-0.003
T. teniotis	105768.5	**0.005**	-0.338	128139.7	**<0.001**	-0.620	30018.3	**<0.001**	0.620	73994.8	0.576	0.064
**total activity**	91961.0	0.154	-0.163	108203.9	**<0.001**	-0.368	49954.1	**<0.001**	0.368	77220	0.838	0.024

Table S3 The results of the Pearson test between species/group activity (sequences/recording hour) and the land cover type of the 1km buffer zone.

	artificial			agriculture			forest		
	**S**	**p**	**rho**	**S**	**p**	**rho**	**S**	**p**	**rho**
B. barbastella	18816.1	0.038	0.283	25022	0.740	0.046	26977	0.839	-0.028
Myotis spp.	56864	0.053	0.223	54254	**0.024**	0.258	91495	**0.029**	-0.251
Nyctaloid all	60847	0.146	0.168	84921.9	0.146	-0.161	63832	0.273	0.127
Pipistrelloid high frequency	59484	0.106	0.187	69534	0.672	0.049	78833	0.505	-0.078
Pipistrelloid low frequency	75020.0	0.655	0.051	92911.3	0.126	-0.175	67222.1	0.190	0.150
Pipistrelloid unidentified	26882	0.860	-0.025	27782	0.672	-0.059	24632	0.661	0.061
Plecotus spp.	9308.3	**0.003**	0.426	9135.3	**0.002**	0.437	23295	**0.002**	-0.437
R. ferrumequinum	62257	0.199	0.149	68997	0.626	0.057	75212	0.809	-0.028
Rhinolophus other than R.fer	67102	0.478	0.083	77833	0.583	-0.064	66726	0.451	0.088
T. teniotis	28998.2	**<0.001**	0.633	44115.5	**<0.001**	0.442	113313.4	**<0.001**	-0.433
**total activity**	48330	**<0.001**	0.389	64972	0.118	0.178	93168	0.119	-0.178

Table S4 The results of the Pearson test between species/group activity (sequences/recording hour) and the land cover type of the 500 m buffer zone.

	artificial			agriculture			forest		
	**S**	**p**	**rho**	**S**	**p**	**rho**	**S**	**p**	**rho**
B. barbastella	2638.6	0.331	0.195	2182.7	0.089	0.334	4369.3	0.089	-0.334
Myotis spp.	8454.5	0.655	0.075	7230.5	0.208	0.209	11047.5	0.208	-0.209
Nyctaloid all	7189.1	0.198	0.213	9094.3	0.977	0.005	9183.7,	0.977	-0.005
Pipistrelloid high frequency	6996.3	0.157	0.234	11472	0.122	-0.255	6805.6	0.122	0.255
Pipistrelloid low frequency	10656.2	0.635	-0.079	10339.8	0.778	-0.047	9420.2	0.778	0.047
Pipistrelloid unidentified	3621.8	0.965	0.009	3951.3	0.681	-0.081	3356.7	0.681	0.081
Plecotus spp.	920.44	**0.007**	0.545	920.44	**0.007**	0.545	3127.6	**0.007**	-0.545
R. ferrumequinum	8354.9	0.609	0.086	8504.4	0.679	0.069	9773.6	0.679	-0.069
Rhinolophus other than R.fer	8354.9	0.609	0.086	9021.4	0.939	0.013	9256.6	0.939	-0.013
T. teniotis	2659.5	**<0.001**	0.731	5969.9	**0.013**	0.396	13790.1	**0.013**	-0.396
**total activity**	6691.1	0.045	0.323	7953.8	0.234	0.195	11806	0.234	-0.195

Table S5 Mean activity (sequence/hour) per species/group in each season and each pond. Mean is calculated from both sites (close and far from water).

Pond	season		*Barbastellus barbastella*	Chiroptera unidentified	*Myotis* spp.	Nyctaloid	Pipistrelloid high frequency	Pipistrelloid unidentified	Pipistrelloid low frequency	*Pipistrellus hanaki*	*Plecotus* spp.	*Rhinolophus ferrumequinum*	*Rhinolophus* other species	*Tadarida teniotis*	Total activity
**Brentanou**	spring	average	0.351	2.362	0.093	15.649	0.694	0.081	43.731			0.041	0.044	1.123	64.167
		stdev	*0.428*	*1.912*	*0.131*	*9.229*	*0.856*	*0.072*	*12.227*			*0.081*	*0.050*	*1.309*	*15.401*
	summer	average	0.415	3.575	0.207	5.026	3.073	13.627	39.845			0.052	0.000	3.523	69.342
		stdev	*0.440*	*2.858*	*0.147*	*2.272*	*3.124*	*17.778*	*9.013*			*0.073*	*0.000*	*0.879*	*23.686*
**Krini**	autumn	average	0.014	2.918	0.101	0.699	7.533	2.428	24.591		0.013	0.040	0.026	0.223	38.586
		stdev	*0.035*	*2.748*	*0.247*	*0.780*	*8.639*	*2.570*	*27.078*		*0.032*	*0.044*	*0.063*	*0.156*	*29.595*
	spring	average	0.210	4.489	4.041	0.305	3.226	1.519	29.789		0.015	0.000	0.031	0.070	43.695
		stdev	*0.515*	*8.617*	*9.393*	*0.239*	*5.260*	*1.862*	*53.147*		*0.036*	*0.000*	*0.048*	*0.061*	*70.210*
	summer	average	0.527	12.550	3.187	2.932	6.226	3.310	88.105		0.000	0.077	0.013	0.379	117.306
		stdev	*1.205*	*22.491*	*7.379*	*3.681*	*8.512*	*6.793*	*121.847*		*0.000*	*0.070*	*0.037*	*0.817*	*156.572*
	winter	average	0.000	0.017	0.000	0.017	0.888	2.842	0.171		0.000	0.000	0.000	0.079	2.760
		stdev	*0.000*	*0.035*	*0.000*	*0.033*	*0.386*	*3.304*	*0.169*		*0.000*	*0.000*	*0.000*	*0.078*	*3.347*
**Omalos**	autumn	average		0.000	2.941	0.091	45.527	2.878	5.631	27.081	0.152	0.079	0.114	63.156	147.650
		stdev		*0.000*	*4.528*	*0.130*	*46.435*	*2.154*	*10.349*	*44.914*	*0.180*	*0.111*	*0.152*	*81.400*	*76.487*
	spring	average		0.000	6.875	0.295	30.366	6.029	2.717	12.598	0.034	9.869	0.021	27.257	96.063
		stdev		*0.000*	*11.846*	*0.538*	*37.316*	*9.841*	*5.177*	*16.014*	*0.047*	*15.117*	*0.039*	*20.621*	*97.920*
	summer	average		0.017	3.224	3.975	7.605	1.212	43.289	42.515	0.181	0.177	0.063	204.703	306.960
		stdev		*0.040*	*2.915*	*5.311*	*11.641*	*1.896*	*23.203*	*49.046*	*0.158*	*0.162*	*0.097*	*163.760*	*165.406*
	winter	average		0.000	0.035	0.000	36.747	1.853	0.069	0.479	0.000	0.000	0.000	20.209	59.392
		stdev		*0.000*	*0.049*	*0.000*	*39.846*	*1.057*	*0.098*	*0.385*	*0.000*	*0.000*	*0.000*	*6.377*	*34.764*
**Vrelli**	autumn	average	0.023	7.560	0.051	10.654	13.897		47.346			0.360	0.071	3.466	83.429

	stdev	*0.057*	*11.440*	*0.080*	*9.287*	*19.910*		*81.767*		*0.572*	*0.175*	*5.533*	*107.851*
spring	average	0.152	24.031	0.591	3.565	8.286	0.896	46.456		1.593	0.130	25.128	110.080
	stdev	*0.108*	*14.061*	*0.965*	*2.267*	*6.358*		*23.724*		*3.695*	*0.124*	*41.471*	*51.054*
summer	average	0.100	13.671	0.139	12.833	9.614	11.786	111.886		0.113	0.035	0.723	151.079
	stdev	*0.059*	*12.088*	*0.206*	*21.952*	*17.072*		*64.816*		*0.135*	*0.054*	*0.591*	*80.645*
winter	average	0.000	0.103	0.205	0.093	4.413		0.106		0.000	0.027	0.360	5.306
	stdev	*0.000*	*0.160*	*0.414*	*0.166*	*4.480*		*0.072*		*0.000*	*0.066*	*0.667*	*4.363*
